# Overcoming a T‐ALL order: A comprehensive study linking genomics to clinical outcomes

**DOI:** 10.1002/hem3.70027

**Published:** 2024-10-13

**Authors:** Yizhou Huang, Charles E. de Bock

**Affiliations:** ^1^ Children's Cancer Institute Lowy Cancer Research Centre Randwick New South Wales Australia; ^2^ School of Clinical Medicine, Faculty of Medicine, UNSW Sydney Sydney New South Wales Australia

Acute lymphoblastic leukemia (ALL) remains a leading success story of how modern therapies have improved patient outcomes from less than 10% survival rate in the 1950s to exceeding 90% today. This has been in part from the decades of research in the optimal use of chemotherapeutics, and, for B‐cell ALL (B‐ALL), the implementation of risk stratification based on clinical factors (e.g., age and peripheral blood cell counts), minimal/measurable residual disease (MRD), and cytogenetics (favorable, neutral, or unfavorable). However, for T‐cell ALL (T‐ALL), risk stratification is currently only based on MRD levels at the end of induction and again at the end of consolidation therapy with genomics and cytogenetics not considered prognostic factors in treatment decision‐making.^1^ In an effort to include genomics into the risk stratification for T‐ALL, a new study led by Charles Mullighan and David Teachey^2^ has now been published as a landmark analysis of 1300 uniformly treated T‐ALL cases that, for the first time, not only defines a total of 15 discrete genetic subtypes but also links them to clinical outcomes.

## EXPANDING THE GENETIC SUBTYPES OF T‐ALL AND MAPPING THEIR CELL OF ORIGIN

**Figure 1:**
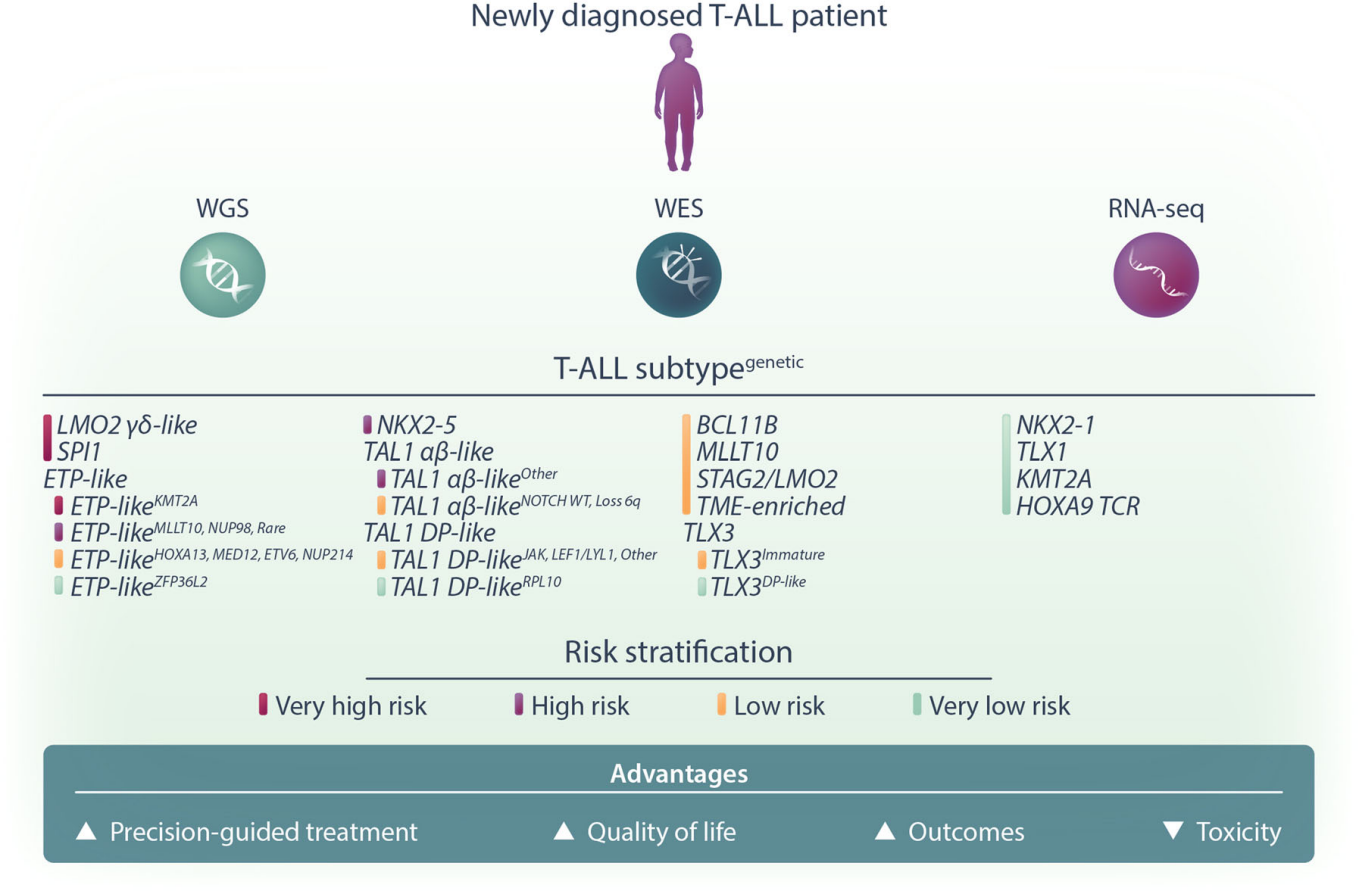
**Future prospects for risk stratification in newly diagnosed T‐ALL patients.** Through comprehensive genomic approaches such as whole genome sequencing (WGS), whole exome sequencing (WES), and transcriptome sequencing (RNA‐Seq), T‐ALL patients can be classified into one of 15 newly identified genetic subtypes (denoted in uppercase). Further stratification into four risk categories—very high, high, low, or very low—can then be determined using additional genetic and clinical factors. This refined classification will enhance patient management by guiding precision‐based treatments and potentially reducing chemotherapy toxicity through de‐escalation for very low‐risk cases. Ultimately, this approach aims to improve both the quality of life and outcomes for T‐ALL patients.

This new study integrates whole genome sequencing (WGS), whole exome sequencing (WES), and whole transcriptome sequencing data to expand the classification of T‐ALL into a total of 15 different subtypes (Figure 1). The most significant variation from the current classification is the definition of two new subtypes, including a new early T‐cell precursor (ETP)‐like ALL subtype and an LMO2 γδ‐like subtype—both of which have a diverse set of genetic alterations. Of the many genetic alterations, an interesting discriminator is the *KMT2A* fusions present in the ETP‐like subtype being mostly *KMT2A::AFDN* fusion, while the non‐ETP subtypes exclusively have *KMT2A::MLLT1* fusion. The authors also compared the gene expression signatures of all 15 subtypes with normal hematopoietic and T‐cell development cell stages. They found that the different T‐ALL subtypes mapped across the entire continuum of T‐cell development, supporting the hypothesis that each subtype represented a “frozen” stage of cellular differentiation. In the case of the ETP‐like subtype, despite the heterogenous genetic drivers, the most likely cell of origin was found to be hematopoietic stem and progenitor cells (HSPC).

## NOTCH ALL THE SAME?

It will come as no surprise that this study confirms the high frequency of recurrent *NOTCH1* mutations (69% of cases) in T‐ALL, second only to *CDKN2A* alterations (71% of cases), with the majority being coding sequence mutations that lead to activation of NOTCH1 signaling. However, this study also found rare single‐nucleotide variants (SNV) within intron 28 of the *NOTCH1* gene which generated a new splice acceptor site and resulted in a 43 amino acid insertion between the heterodimerization (HD) domain and the transmembrane (TM) domain of NOTCH1. Functionally, this new mutation drove the “strongest” NOTCH1 signaling compared to other *NOTCH1* mutations when tested in a luciferase reporter‐based system. Interestingly, while *NOTCH1* mutations are often considered to be favorable for prognosis, these intronic SNV mutations are associated with poor patient outcomes. This follows an independent study showing recurrent *NOTCH1* gene fusions occur in very high‐risk cases of related T‐cell lymphoma.^3^ These two studies continue to support the ongoing need for safe and effective NOTCH1 inhibitors. In the case of intronic SNV‐generated novel 43 amino acid insertion, one potential option would be to develop immune‐based therapies targeting this T‐ALL‐specific neoepitope and prevent “on‐target off‐tumor” side effects that plague other NOTCH1 inhibitors such as the broad‐spectrum gamma‐secretase inhibitors.

## HIGH FREQUENCY OF ENHANCER HIJACKING

One of the advantages of using WGS in this study was the discovery of enhancer hijacking‐mediated oncogene activation present in over 70% of T‐ALL cases. This is where enhancers are juxtaposed to oncogenes through a range of different chromosomal events including translocation, inversions, and chromothripsis, with hijacking of the T‐cell receptor (TCR) enhancer being the most frequent event.

## IMPROVED RISK STRATIFICATION FOR T‐ALL

What sets this study apart from previous landscape sequencing studies is the link between genomic features and clinical outcomes. In this study, all patients sequenced were uniformly treated, providing a platform to generate a multivariable model for risk stratification. This is a major step forward in managing T‐ALL patients, as ETP‐ALL subtype is, to date, the only defined T‐ALL subtype that clinicians might consider with respect to treatment decision‐making. In this study, the authors risk‐stratified patients based on their genomic features, altered genes, and dysregulated pathways, resulting in four broad risk groups of “very high risk,” “high risk,” “low risk,” and “very low risk” (Figure 1). They were then further subdivided based on their Day 29 MRD status for a total of eight risk groups. One interesting finding is that, for patients with *KMT2A* rearrangements, the subtype context is critical for the clinical outcome and risk grouping. For example, a patient with ETP‐like and *KMT2A* subtype T‐ALL is classified as “very high risk,” while a patient with non‐ETP‐like and *KMT2A* subtype is classified as “low risk.” The *SPI1* subtype is also interesting because while classified as “very high risk,” they are more likely to be MRD negative. Their poor outcome is in part due to the development of secondary malignancies that also harbor the *SPI1* fusion. Recently, it was found that T‐ALL cases harboring *SPI1* fusions are highly sensitive to dasatinib.^4^ Therefore, with this new study showing the high propensity for patients developing secondary malignancies that also carry the *SPI1* fusion, the use of dasatinib might be considered earlier in treatment, albeit it remains to be seen whether the use of kinase inhibitors will change the trajectory of both the T‐ALL clone and the secondary malignancy.

So how does this new study change the management of newly diagnosed T‐ALL patients and can this new risk stratification be used prospectively? Pleasingly, the authors developed these risk models with clinical translation in mind such that new T‐ALL patients can be stratified using a focused selection of features. Therefore, if applied prospectively, patients classified as “very high risk” could be fast‐tracked to bone marrow transplantation. Conversely, for “low risk” patients (e.g., MRD‐negative, non‐ETP‐like *KMT2A* subtype), either intensified chemotherapy could be de‐escalated or a treatment‐free interval could be introduced. These approaches may help manage the toxic side effects of chemotherapy, potentially improving the quality of life without adversely affecting clinical outcomes (Figure 1).

## CONCLUDING REMARKS

This study provides a wealth of new information for researchers studying the biology of T‐ALL and clinicians to help manage patients when next‐generation sequencing data are available. It was not long ago when in 2017 at a small conference in Leuven, Belgium, Charles Mullighan presented transcriptome and WES data on a total of 264 T‐ALL cases.^5^ One question that was asked at the end of the presentation was whether sequencing more patients would add any new information. The reply from Charles was “Absolutely. We are only beginning to scratch the surface*.*” We are confident that if either of the lead authors were asked the same question again, their response would likely remain unchanged.

## AUTHOR CONTRIBUTIONS

Both Yizhou Huang and Charles E. de Bock conceptualized and cowrote the article. Both authors agreed to the final version.

## CONFLICT OF INTEREST STATEMENT

The authors declare no conflicts of interest.

## FUNDING

Yizhou Huang and Charles E. de Bock are both supported by the National Health and Medical Research Council (NHMRC) Ideas Grant 2029411. No funding was received for this publication.

## Data Availability

Data sharing is not applicable to this article as no data sets were generated or analyzed during the current study.
